# Phylogenetic and functional characterisation of the *Haemophilus influenzae* multidrug efflux pump AcrB

**DOI:** 10.1038/s42003-019-0564-6

**Published:** 2019-09-13

**Authors:** Martijn Zwama, Akihito Yamaguchi, Kunihiko Nishino

**Affiliations:** 1 Department of Biomolecular Science and Regulation, Institute of Scientific and Industrial Research, Osaka University, Ibaraki, Osaka 567-0047 Japan; 2 Laboratory of Cell Membrane Biology, Institute of Scientific and Industrial Research, Osaka University, Ibaraki, Osaka 567-0047 Japan

**Keywords:** Biochemistry, Microbiology, Antimicrobial resistance

## Abstract

Multidrug resistance in Gram-negative bacteria can arise by the over-expression of multidrug efflux pumps, which can extrude a wide range of antibiotics. Here we describe the ancestral *Haemophilus influenzae* efflux pump AcrB (AcrB-Hi). We performed a phylogenetic analysis of hundreds of RND-type transporters. We found that AcrB-Hi is a relatively ancient efflux pump, which nonetheless can export the same range of antibiotics as its evolved colleague from *Escherichia coli*. AcrB-Hi was not inhibited by the efflux pump inhibitor ABI-PP, and could export bile salts weakly. This points to an environmental adaptation of RND transporters. We also explain the sensitivity of *H. influenzae* cells to β-lactams and novobiocin by the outer membrane porin OmpP2. This porin counterbalances the AcrB-Hi efflux by leaking the drugs back into the cells. We hypothesise that multidrug recognition by RND-type pumps is not an evolutionarily acquired ability, and has been present since ancient promiscuous transporters.

## Abstract (web summary)

Zwama et al. demonstrate that the AcrB efflux pump from *H. influenzae* (AcrB-Hi) is phylogenetically far removed from AcrB in *E. coli* but show similar specificity. They also show that efflux is counteracted by leakage of some antibiotics back into the bacterial cell through the OmpP2 channel which can explain *H. influenzae* sensitivity to these compounds.

## Introduction

H*aemophilus influenzae* Type b (Hib) is a clinically relevant pathogenic Gram-negative bacterium that causes several invasive diseases (especially in young children), including meningitis and pneumonia^1,2^. Although conjugate vaccinations significantly decreased the number of Hib-related infectious diseases, Hib still causes illnesses all around the world^3^. Today, antimicrobial-resistant pathogens form a great threat to modern health worldwide^4^. β-lactams (such as ampicillin or third-generation cephalosporins) are often used as first- and second-line drugs to treat Hib infections^5^, as until recently Hib did not possess β-lactamase genes. However, today, β-lactam resistance has been observed in clinical Hib isolates: BLNAR (Beta-Lactamase Non-producing Ampicillin Resistance, caused by TEM-1 and ROB-1 β-lactamases^6^) and BLPAR (Beta-Lactamase-Producing Ampicillin Resistance, caused by lower affinities in PBPs by amino acid substitutions^7,8^) have become serious threats in Hib chemotherapy^9^. Resistance to a single class of antibiotics can be caused by one resistance factor, such as the production of β-lactamases or altered PBPs^10,11^. On the other hand, multidrug resistance (MDR) in Gram-negative bacteria can be caused solely by the overexpression of antibiotic efflux pumps. These pumps render several or all classes of antibiotics commercially available today ineffective^12^. Such a multidrug efflux pump (called AcrB) belonging to the resistance-nodulation-division (RND) superfamily is expressed in *H*. *influenzae* cells. AcrB is part of the tripartite efflux system AcrAB-TolC that spans both the inner and outer membrane of the Gram-negative cells, directly facilitating the efflux of antibiotics from the cytoplasm or periplasm out of the cell. *H. influenzae* AcrB (AcrB-Hi) is an efflux pump that was shown to expel several antibiotics and dyes similar to *Escherichia coli* AcrB (AcrB-Ec)^13^. Although this efflux pump has been described previously to cause macrolide resistance^14^, relatively little is known about the role of AcrB in Hib drug resistance, in addition to its large outer membrane protein OmpP2.

Here we show that AcrB-Hi is a relatively ancient pump, phylogenetically far removed from AcrB-Ec. Despite their relatively low genetic relation and homology, AcrB-Hi can transport similar compounds as its evolved colleague AcrB-Ec. However, we also found differences in substrate specificities. We show that AcrB-Hi can export bile salts only weakly. Furthermore, AcrB-Hi is uninhibited by pyridopyrimidine-derived efflux pump inhibitor (EPI) ABI-PP, which completely inhibits AcrB-Ec. We observe that the defence mechanism of *H. influenzae* is counterbalanced by the leakage of antibiotics through the OmpP2 outer membrane channel back into the bacterial cells, providing a molecular explanation as to why Hib cells are sensitive to certain antibiotics. Our data suggest that ancestral less-efficient and nonspecific RND transporters have evolved to become efficient and more specific transporters, with physiologically relevant specificities. From our data, we hypothesise that multidrug recognition by multidrug efflux pumps did not increase during evolution and that the ability to export chemically different compounds is an intrinsic property since ancient transporters.

## Results

### *Haemophilus influenzae* AcrB is an ancestral RND efflux pump

**Fig. 1:**
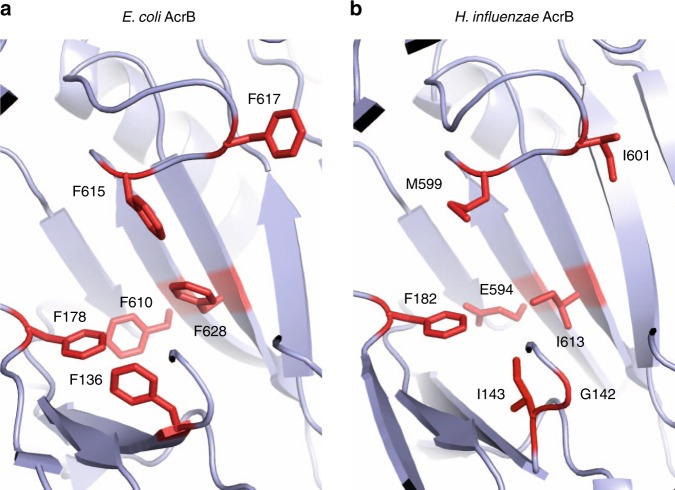
The hydrophobic pits of AcrB-Ec and AcrB-Hi. **a** The phenylalanine residues in the hydrophobic trap in the crystal structure of AcrB-Ec (PDB identification code 3AOA). The six residues are Phe136, Phe178, Phe610, Phe615, Phe617, and Phe628 and this pit is therefore also described as the phenylalanine rich pit. **b** The corresponding residues in the hydrophobic trap of AcrB-Hi, based on the crystal structure of AcrB-Ec (PDB accession code 3AOA) according to homology modelling. These residues are Gly142 or Ile143, Phe182, Glu594, Met599, Ile601, and Ile613. The ABI-PP inhibitor-bound pit models can be found in Supplementary Fig. 6

To investigate the RND efflux pump AcrB expressed in *H. influenzae* (AcrB-Hi), we decided to compare the pump to the probably most studied RND multidrug efflux pump AcrB from *E. coli* (AcrB-Ec). AcrB-Ec is known as a promiscuous efflux pump with an extremely wide efflux range of many structurally unrelated compounds, which include dyes, antibiotics, bile salts, and detergents^15,16^. Although the substrate specificity of RND transporters is generally wide, the pumps are not non-specific, as paralogues and orthologues (such as AcrB vs. AcrD, and MexB vs. MexY)^17–20^ differ in substrate recognition depending on the physiochemical properties of the compounds^21–24^. We found that *acrB* is the only RND transporter gene (HI0895) in the *Haemophilus influenzae* Rd KW20 genome with a translated length of 1032 amino acids (AcrB-Ec has 1049 amino acids). Initial amino acid sequence alignment shows the two proteins share 31% identity, 52% similarity and 4% gaps (Supplementary Fig. 1). The hydrophobic pit (or hydrophobic trap) of AcrB-Ec holds six phenylalanine residues^25^ (the Phe-rich pit), namely Phe136, Phe178, Phe610, Phe615, Phe617, and Phe628. These correspond to Gly142, Phe182, Glu594, Met599, Ile601, and Ile613 in AcrB-Hi. Our homology model suggests Phe136 may correspond to Ile143 (Fig. 1). Most of these residues are hydrophobic (Ile143, Met599, Ile601, and Ile613), one is aromatic (Phe182), and one is charged (Glu594). Between AcrB-Hi and AcrB-Ec, the transmembrane domain is mostly preserved (40% identity and 67% similarity), of which transmembrane helix 4 (TM4) is the most preserved (59% identity and 89% similarity).

**Fig. 2:**
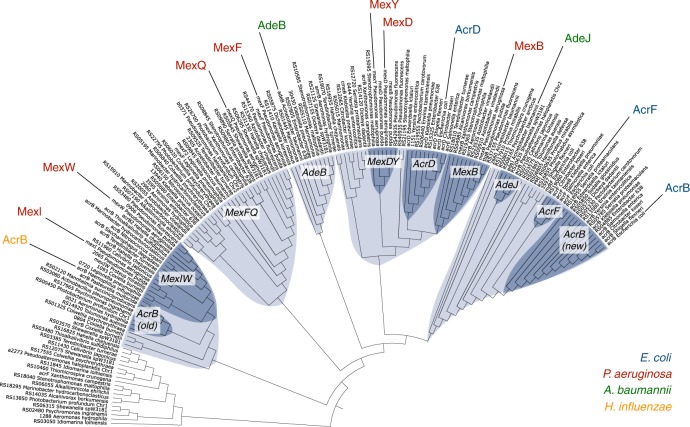
Phylogenetic tree of a selection of RND transporters. Phylogenetic relationship between several homologous RND transporters (~160) from gammaproteobacteria analysed by multiple sequence alignment, branched into several clusters. One of the two latest branched clusters harbour *E. coli* AcrB-Ec (blue, right), AcrF (blue) and *A. baumannii* AdeJ (green), and the other cluster *P. aeruginosa* MexB, MexD, MexY (red) and *E. coli* AcrD (blue). Two genetically further distanced clusters contain *A. baumannii* AdeB (green) and *P. aeruginosa* MexB and MexD (red), respectively. Then *H. influenzae* AcrB (yellow, left) and *P. aeruginosa* MexI and MexW (red) are situated in genetically further removed phylogenetic clusters. Clusters and sub-clusters are highlighted in blue and dark blue, respectively. The complete tree of all 393 analysed RND genes can be found in Supplementary Fig. 2. Branch lengths can be seen in Supplementary Fig. 3

The next step in comparing AcrB-Hi with AcrB-Ec was to collect gene sequences from multiple Gram-negative bacteria homologous to the *acrB*-Hi and *acrB*-Ec genes. We collected 393 homologous genes from 51 gammaproteobacteria and analysed them by multiple sequence alignment (Supplementary Data 1). Genes could be classified into three major clusters: MDR (including clusters for *mdtB* and *mdtC*), heavy metal (separated into clusters for *cusA* and *czcA*) and other (including triclosan pump *triC*^26^) (Supplementary Figs. 2, 3). MDR transporters comprised about half (Supplementary Fig. 2) and are shown in Fig. 2 (with the exclusion of the heteromeric MdtBC-like systems). The phylogenetic tree (Fig. 2) shows that *acrB*-Hi is phylogenetically far removed from *acrB*-Ec. AcrB-Ec is one of the latest evolved pumps in the phylogenetic tree and is most closely related to homologue AcrF from *E. coli* and orthologue AdeJ from *Acinetobacter baumannii*. These transporters then share a branching point with a cluster containing *E. coli* AcrD and *Pseudomonas aeruginosa* MexB, MexD, and MexY. Further genetically distanced genes belong to other clusters; firstly harbouring *A. baumannii* AdeB, and then *P. aeruginosa* MexF^27^ and MexQ. Even further genetically distanced is a cluster separated into two genetic sub-clusters; one containing *P. aeruginosa* MexI^28^ and MexW^29^ and the other one AcrB-Hi. AcrB-Hi therefore appears to be a relatively ancestral efflux pump, closely related to the AcrB efflux pump from zoonotic pathogen *Pasteurella multocida*, and other RND pumps from e.g. pathogenic Q-fever causing *Coxiella burnetii* and marine bacterium *Photobacterium profundum* (Fig. 2).

### AcrB-Hi is an active promiscuous MDR efflux pump

As we found that AcrB-Hi is phylogenetically distant from AcrB-Ec, we wanted to test for its substrate specificity and compare it to AcrB-Ec. Table 1 shows the minimal inhibitory concentration (MIC) data for 15 different compounds, including planar aromatic cations (PACs), low-molecular-mass drugs (LMMDs), high-molecular-mass drugs (HMMDs) and β-lactams (including penicillins, cephalosporins, carbapenems, and monobactams). The *acrRAB*-cluster from the *H. influenzae* strain Rd KW20 genome was cloned into plasmids (pBAD33*acrRAB*HiT, Supplementary Table 1) and transformed into *E. coli* MG1655∆*acrAB* cells. AcrB-Hi and adapter protein AcrA-Hi were functioning with *E. coli* TolC (TolC-Ec). AcrB-Ec and AcrB-Hi expression levels were similar (Supplementary Fig. 4). Compared to AcrB-Ec, AcrB-Hi is a relatively less-efficient transporter, with MIC values identical to or lower than AcrB-Ec. Nonetheless, it was able to transport the same compounds tested as AcrB-Ec.

**Table 1:** Minimal inhibitory concentrations (MIC) for several antibiotics for AcrB-Ec and AcrB-Hi expressing *E. coli* MG1655 cells

Genotype	Plasmid	Minimal inhibitory concentration (MIC, µg mL^−1^)														
		PAC			LMMD			HMMD		β-lactam						
										Penicillins			Cephalosporins		Carbapenem	Monobactam
		EtBr	R6G	CV	MINO	DEQ	KAN	EM	NOV	CLX	BPEN	MET	CEFP	CTX	DORI	AZT
∆*acrB*	pBAD33	16	16	2	1	1.6	8	4	8	2	16	16	0.016	0.016	0.03125	0.0625
∆*acrB*	pBAD33*acrB*his	**256**	**>512**	**16**	**4**	**100**	*8*	**64**	**512**	**128**	**32**	**>512**	**8**	*0.016*	0.0625	*0.0625*
∆*acrAB*	pBAD33*acrRAB*HiT	**128**	**256**	**16**	2	**50**	*8*	**32**	**64–128**	**64**	**16–32**	**128**	**1**	*0.016*	0.0625	*0.0625*

Liquid MIC values determined by a twofold dilution method, where cells are grown in LB broth supplemented with drugs as described in Methods. Bold indicates a significant at least two dilutions increase in MIC compared to KO cells, italic indicates the same MIC as KO cells. Data based on at least two independent experiments. An overview of relative MICs between AcrB-Ec and AcrB-Hi can be seen in Supplementary Fig. 5

*PAC* planar aromatic cation, *LMMD* low-molecular-mass drug, *HMMD* high-molecular-mass drug, *EtBr* ethidium bromide, *R6G* rhodamine6G, *CV* crystal violet, *MINO* minocycline, *DEQ* dequalinium, *KAN* kanamycin, *EM* erythromycin, *NOV* novobiocin, *CLX* cloxacillin, *BPEN* benzylpenicillin, *MET* methicillin, *CEFP* cefcapene pivoxil, *CTX* ceftriaxone, *DORI* doripenem, *AZT* aztreonam, *acrBhis* his-tagged *E. coli* *acrB*, *acrRABHiT H. influenzae acrR*, *acrA* and his-tagged *acrB*

PACs, erythromycin (EM), antiseptic dequalinium (DEQ) and the penicillin cloxacillin (CLX) show a relatively high MIC compared to AcrB-Ec, showing only a lowering in MIC of up to one dilution (while also being significantly more active than *acrB*-deficient MG1655 cells). At the same time, HMMD aminocoumarin novobiocin (NOV), penicillin methicillin (MET) and cephalosporin cefcapene pivoxil (CEFP) show a somewhat lower MIC when AcrB-Hi was expressed, with a lowering of the MIC values by 2−3 dilutions compared to AcrB-Ec (although also still being significantly higher than *acrB*-deficient cells). On the other hand, compared to *acrB* knockout (KO) cells, CEFP shows the relatively highest MIC of six dilutions higher than KO cells. It appears that the antibiotic specificity of AcrB-Hi is very similar to AcrB-Ec, although depending on the compound, the MIC is either similar or significantly lower (Supplementary Fig. 5). For both AcrB-Ec and AcrB-Hi expressing cells, third-generation cephalosporin ceftriaxone (CTX), monobactam aztreonam (AZT) and aminoglycoside kanamycin (KAN) show identical MIC values as the *acrB*-deficient cells. LMMD tetracycline minocycline (MINO), carbapenem doripenem (DORI) and penicillin benzylpenicillin (BPEN) only show a small elevation of the MIC compared to *acrB* KO cells. (Supplementary Fig. 5).

We were particularly interested to check the AcrB-Hi efflux ability of β-lactams, as these antibiotics are often used as a first-line treatment against *H. influenzae*-induced infectious diseases^5^. AcrB-Hi can export penicillin (CLX, BPEN and MET), cephalosporin (CEFP) and slightly carbapenem (DORI) antibiotics (Table 1). Relatively to AcrB-Ec, from these antibiotics, AcrB-Hi-expressing cells showed a significantly high MIC for CLX and least significantly for MET (Supplementary Fig. 5). In absolute terms, for AcrB-Hi expressing cells, the highest MIC values were observed for MET (128 µg mL^−1^) and CLX (64 µg mL^−1^). It must be noted that AcrB-Hi was expressed within *E. coli* cells; it is therefore possible that the unnatural environment of *E. coli* played a role in the relatively lower MICs; however, AcrB-Ec and AcrB-Hi were expressed in similar abundance (Supplementary Fig. 4). The presence of AcrR-Hi and TolC-Ec is discussed in the Discussion section. Nonetheless, the MIC values for AcrB-Ec- and AcrB-Hi-expressing cells were either identical (for CV), just slightly lower (for DEQ, CLX, EtBr and EM) or significantly lower (for R6G, NOV, and MET) (Supplementary Fig. 5), pointing to a divergent resistance caused by intrinsic differences between these two transporters. Despite the relatively distant phylogenetic relationship with AcrB-Ec, AcrB-Hi too is a promiscuous MDR efflux pump.

### AcrB-Hi is uninhibited and exports bile salts weakly

The phylogenetic tree (Fig. 2) shows that AcrB-Hi is a relatively ancestral MDR pump which—despite the low similarity with AcrB-Ec—could transport the same antibiotics and dyes (Table 1). We set out to investigate potential differences between AcrB-Ec and AcrB-Hi. As RND pumps have a physiological function in bacterial cells besides being MDR efflux systems^30^, and because AcrB-Ec seemed to be evolved significantly later, we wanted to investigate the efflux ability of AcrB-Hi for AcrB-Ec biologically relevant compounds: bile salts. These toxic compounds are present in the natural environment of enteric *E. coli* cells, but not of *H. influenzae* cells. Bile salts, including cholic acid (CHO) and deoxycholic acid (DEOX), are excellent substrates of AcrB-Ec, but not of all MDR RND transporters^17^. Table 2 shows the MIC results for bile salt (mixture), CHO, DEOX and indole for AcrB-Ec- and AcrB-Hi-expressing cells. AcrB-Hi could not export the bile salts efficiently, with MIC values of only one dilution higher than the *acrB* KO cells, while cells expressing AcrB-Ec had MIC values 16- to 32-fold higher than the *acrB* KO cells, for CHO, bile salts mixture, and DEOX, respectively. The MIC values for AcrB-Hi- and AcrB-Ec-expressing cells for bile salts and deoxycholic acid were 2000 vs. >16,000 µg mL^−1^ (>8-fold) and 1600 vs. >25,600 µg mL^−1^ (>16-fold), with *acrB* KO MICs as 1000 and 800 µg mL^−1^, respectively. The MIC values for AcrB-Ec could be even higher, as the tested compound concentrations reached its limit (Table 2, Supplementary Fig. 5). A recent crystal structure of efflux pump regulator RamR cocrystallised with bile salts shows that bile salts can bind in the voluminous binding pocket of the regulator protein by forming hydrogen bonds^31^ rather than by π−π interactions used to bind PACs^32^. The hydrophobic pit in AcrB-Hi does not comprise a Phe-rich cluster (Fig. 1, Supplementary Fig. 1); however, it is probably not the Phe-residues that recognise bile salts, because bile salt molecules do not contain aromatic moieties. AcrB-Hi also did not seem to export aromatic indole, with an identical MIC value as the *acrB* KO cells, although the MIC for AcrB-Ec was only (at least) one dilution higher compared to the KO cells (Table 2).

**Table 2:** Minimal inhibitory concentrations (MIC) for bile salts for AcrB-Ec and AcrB-Hi expressing *E. coli* MG1655 cells

Genotype	Plasmid	Minimal inhibitory concentration (MIC, µg mL^−1^)			
		Bile salts			Other
		Mix	CHO	DEOX	Indole
∆*acrB*	pBAD33	1000	6400	800	512
∆*acrB*	pBAD33*acrB*his	>16,000	>25,600	>25,600	>512
∆*acrAB*	pBAD33*acrRAB*HiT	**2000**	**12,800**	**1600**	512

Liquid MIC values determined by a twofold dilution method, where cells are grown in LB broth supplemented with drugs as described in Methods. Bold shows an only one dilution higher MIC than KO cells, where bold underlined additionally indicates a significant decrease in MIC for AcrB-Hi compared to AcrB-Ec. Data based on at least two independent experiments. An overview of relative MICs between AcrB-Ec and AcrB-Hi can be seen in Supplementary Fig. 5

*CHO* cholic acid, *DEOX* deoxycholic acid, *acrBhis* his-tagged *E. coli acrB*, *acrRABHiT H. influenzae acrR*, *acrA* and his-tagged *acrB*

**Fig. 3:**
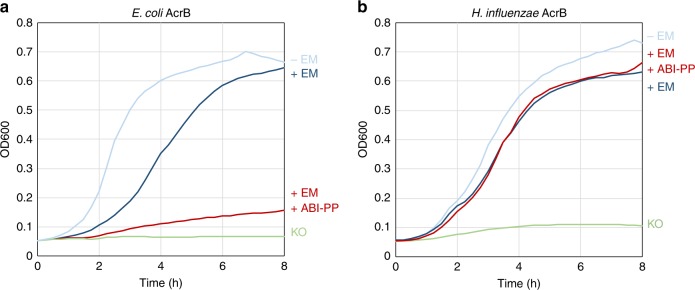
Inhibitory effect of ABI-PP on AcrB-Ec and AcrB-Hi. **a** Efflux pump inhibitor ABI-PP was able to inhibit the efflux activity of AcrB-Ec (red). **b** ABI-PP was unable to inhibit AcrB-Hi (red). Colours light blue and dark blue show AcrB-expressing cells without or with the addition of erythromycin, respectively, and green shows *acrB* knockout cells in the presence of erythromycin. Red shows the growth ability of AcrB-expressing *E. coli* cells with the addition of both erythromycin and ABI-PP. All erythromycin concentrations were 32 µg mL^−1^ and ABI-PP concentrations were 64 µg mL^−1^ (**a**, **b**). EM erythromycin, KO *acrB* knockout. Data shown are one of the results, repeats (at least four) showed similar results

Because the Phe-lacking AcrB-Hi expressed in *E. coli* cells showed very low MICs for bile salts close to KO cells, we additionally wanted to know the potential implications of the deficiency of the Phe-rich hydrophobic trap. The first crystal structure of an RND transporter with an efflux pump inhibitor (EPI) was AcrB-Ec co-crystallised with pyridopyrimidine-derived EPI ABI-PP^25^. ABI-PP was located in the Phe-rich hydrophobic trap in the binding monomer of the trimer, blocking the transport ability of the pump. The Phe-residues were strongly interacting with the aromatic moieties of the inhibitor by π−π interactions, especially with Phe178 and Phe615. Figure 3 shows the growth ability of both AcrB-Hi- and AcrB-Ec-expressing cells in the presence or absence of EM and ABI-PP. AcrB-Ec was completely inhibited by the inhibitor ABI-PP as found before^25^; however, AcrB-Hi was completely unaffected by the EPI and cells showed full viability. When ABI-PP was added in even higher concentrations (double and triple), there was still no effect. Although the presence of the Phe cluster in AcrB-Ec may be important for efficient binding and export of some pit-binding compounds^33,34^ and inhibitors^25,35^, they seem not to be crucial for the export of antibiotics and dyes (Table 1), although they are important for the tight binding of and inhibition by pyranopyridine-derived EPIs (Figs. 1, 3, Supplementary Fig. 6)^25,35^. However, the inability to recognise and export bile salts effectively (Table 2) should perhaps be explained by other regions or differences in the transporter (possibly in the binding pockets^36,37^ or the entrance channels^37,38^).

As we found that AcrB-Hi was not inhibited by ABI-PP and that for certain compounds AcrB-Hi-expressing cells show a divergent MIC pattern compared to AcrB-Ec-expressing cells, we tried to partly mimic the AcrB-Hi pit residues in AcrB-Ec (Supplementary Table 2). We made several single and double Phe-substitutions and checked the MIC for several compounds. Most single and double mutations had a negative effect on the MIC of all tested compounds, which may explain the higher MIC for certain compounds by AcrB-Ec. For example, replacing the Phe136 and Phe628 with Ile or Gly had relatively the biggest effect on R6G and to a lesser extent on EtBr and MET (Supplementary Table 2). Probably multiple differences in residues in both the binding pocket and the hydrophobic pit cause the divergent MIC differences between AcrB-Hi- and AcrB-Ec-expressing cells. Other factors also play a role in substrate recognition. As also seen in Supplementary Table 2, the mutation Phe610Glu in AcrB-Ec (mimicking Glu594 in AcrB-Hi) had little effect on the MIC values, despite the significant change from a hydrophobic aromatic side chain to a negatively charged side chain. Interestingly, the similar mutation Phe610Ala, described too by others^39^ in AcrB-Ec, had a significant negative effect on the MIC values. It is therefore difficult to interpret mutations in the pockets of AcrB, and binding efficiencies alone are not the only determinant of the export of the compounds by AcrB^39^. Additionally, we made mutations in AcrB-Hi to include Phe-residues in the hydrophobic pit to mimic AcrB-Ec to see if the transporter would become sensitive to ABI-PP. In the crystal structure of ABI-PP bound to AcrB, the residues binding most strongly are Phe178, Phe615 and Phe628, and slightly Phe610. Supplementary Fig. 7 shows the results. Phe178 is conserved in AcrB-Hi (Phe182). E594F (resulting in double Phe178 and Phe610) had no effect. M599F (resulting in double Phe178 and Phe615, which strongly interact with ABI-PP in AcrB-Ec) had no effect on ABI-PP inhibition. The introduction of I613F (corresponding to AcrB-Ec Phe628) resulted in a completely inactive transporter. For this reason, we are cautious in interpreting the MIC findings presented in Supplementary Table 2. A more thorough analysis of the binding pockets is needed (by molecular dynamic or chimeric experiments replacing the whole pit or the pockets) in order to better understand the differences in substrate export efficiency and EPI inhibition.

### Antibiotics leak through outer membrane porin OmpP2

AcrB-Hi can actively transport β-lactams (Table 1). Previous research showed that MICs for a β-lactam (carbenicillin), fusidic acid, ciprofloxacin, tetracycline (TET), chloramphenicol (CHL) and norfloxacin (NOR) for Hib cells (expressing AcrB-Hi) were identical to *acrB* KO cells^40^. It was also found that TET, CHL, and NOR were substrates of AcrB-Hi^13^, and here we show that AcrB-Hi can export many other compounds, including a variety of classes of β-lactams (Table 1). β-lactams, including penicillins, are often used as first-line drugs to treat Hib infections^5^, despite that these compounds are actively being effluxed by the *H. influenzae* AcrAB-TolC system (Table 1). The outer membrane (OM) of Hib cells is very permeable^41^, including for β-lactams^42^. The outer membrane of *H. influenzae* contains a wide outer membrane porin OmpP2. We wanted to directly see the possible effect of this large porin on the efflux by AcrB-Hi. We therefore cloned the *ompP2* gene from the *H. influenzae* strain Rd KW20 genome into plasmids (creating pET26b(+)*ompP2*, Supplementary Table 1). Table 3 shows the MICs for ten different compounds. Basically no effect was seen for DEQ, EM and most tested PACs, for both AcrB-Ec- and AcrB-Hi-expressing cells when OmpP2 was introduced. The MICs were identical (for EtBr, DEQ and EM) or decreased one dilution (for R6G). For the cationic CV, however, the MIC values (for AcrB-Ec expressing cells) was decreased by two dilutions. Additionally, for the large molecule NOV the MIC was affected significantly by the expression of OmpP2, as the MIC values (for both AcrB-Ec- and AcrB-Hi-expressing cells) were decreased a significant two dilutions. It must also be noted that for NOV, the expression of OmpP2 had a big effect for all tested concentrations and that therefore the MIC values are within a range and difficult to determine; the significant effect on growth ability under NOV can be observed in Supplementary Fig. 8. Furthermore, a significant decrease in MIC was seen for all the β-lactams CLX, BPEN and MET, with a decrease of MIC ranging between one and three dilutions. Penicillin MET was affected mostly with a decrease in MIC from 128 µg mL^−1^ for AcrB-Hi-expressing cells, to 16 µg mL^−1^ when AcrB-Hi and OmpP2 were both expressed. CLX was also significantly affected, with an MIC decrease from 128 or 64 µg mL^−1^ to 16 µg mL^−1^ when OmpP2 was expressed. A previous study showed that the MICs for other LMMDs (such as TET and CHL) for Hib cells were the same with or without AcrB-Hi^40^ (indicating an influx of these LMMDs through the OM), while the MIC for the LMMD DEQ in our study showed to be unaffected by OmpP2, perhaps because this compound is positively charged. The PACs tested here are also positively charged and also seem to be unaffected by the expression of OmpP2 (Table 3). We did not test for CHL, as this was the selection marker on our pBAD33 constructs, and the tetracycline MINO is not a very good substrate of both AcrB-Ec and AcrB-Hi (Table 1).

**Table 3:** Minimal inhibitory concentrations (MIC) for several compounds for AcrB-Ec, AcrB-Hi and OmpP2 expressing *E. coli* C43(DE3) cells

	Genotype	Plasmid(s)				Minimal inhibitory concentration (MIC, µg mL^-1^)								
						PACs			LMMD	HMMD		β-lactams		
			Proteins expressed									Penicillins		
			AcrB-Ec	AcrB-Hi	OmpP2	EtBr	CV	R6G	DEQ	EM	NOV	CLX	BPEN	MET
	∆*acrB*∆*ompF*	pBAD33 + pET26b(+)				<4	2	8	0.4	2	2	<4	1	2
***AcrB-Ec***	∆*acrB*	pBAD33*acrB*his	✓			32	32	>512	50	64	512	128	4	256
	∆*acrB*∆*ompF*	pBAD33*acrB*his	✓			32	32	>512	50	32	256–512	64	4	256
	∆*acrB*∆*ompF*	pBAD33*acrB*his + pET26b(+)*ompP2*	✓		✓	*32*	**8**	*>512*	*25*	*32*	**128**	**16**	2	**64**
***AcrB-Hi***	∆*acrB*	pBAD33*acrRAB*HiT		✓		32	16	256	50	64	256–512	64	8	128
	∆*acrAB*	pBAD33*acrRAB*HiT		✓		32	8	128–256	50	64	256–512	64	8	128
	∆*acrB*∆*ompF*	pBAD33*acrRAB*HiT + pET26b(+)*ompP2*		✓	✓	*32*	4	*128–256*	*50*	*64*	**<64–128**	**16**	**2**	**16**

Liquid MIC values determined by a twofold dilution method, where cells are grown in LB broth supplemented with drugs as described in Methods. Italic indicates no change in MIC when OmpP2 is expressed, and bold underlined indicates 2 or 3 dilutions decrease in MIC. A check mark (✓) indicates the presence by expression of the respective protein. Data based on at least two independent reproduced experiments

*PAC* planar aromatic cation, *LMMD* low-molecular-mass drug, *HMMD* high-molecular-mass drug, *EtBr* ethidium bromide, *CV* crystal violet, *R6G* rhodamine6G, *DEQ* dequalinium, *EM* erythromycin, *NOV* novobiocin, *CLX* cloxacillin, *BPEN* benzylpenicillin, *MET* methicillin, *acrBhis* his-tagged *E. coli acrB*, *acrRABHiT H. influenzae acrR*, *acrA* and his-tagged *acrB*

We show that OmpP2 is a porin with high permeability, while maintaining a semi-specificity: one cationic compound (CV) and one HMMD (NOV) was affected, while the others were not, and all tested β-lactams seemed to be affected by OmpP2. The effect of OmpP2 on the MICs for our tested compounds can be summarised as β-lactams > NOV > CV> PACs ≧ EM. Large antibiotic EM may also traverse through OmpP2, but at a much slower rate, hence largely not affecting the MIC. The high permeability by the influx through OmpP2 can explain why Hib cells are sensitive to β-lactams and novobiocin, despite the fact that these are actively transported by the AcrB-Hi efflux transporter (Table 1).

## Discussion

**Fig. 4:**
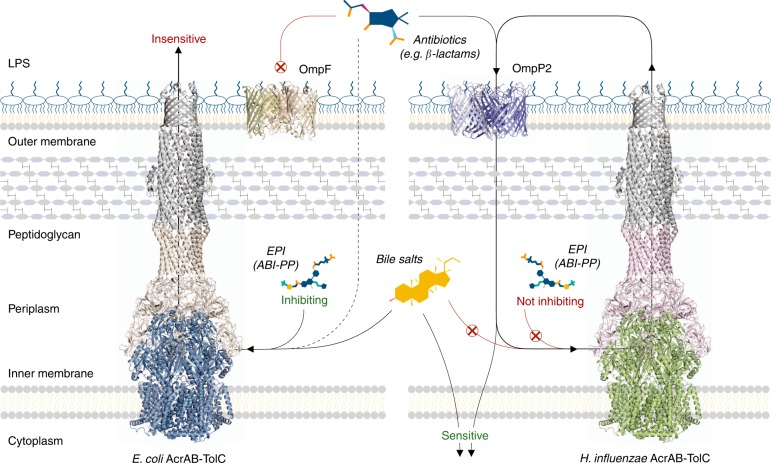
The interplay between efflux pumps and porins from *E. coli* and *H. influenzae*. AcrB efflux pumps can actively transport antibiotics from the periplasm and cytoplasm of bacterial cells, rendering them ineffective. AcrB-Ec is able to export bile salts, which is present in the enteric environment of *E. coli* cells. On the other hand, AcrB-Hi can export bile salts only weakly. Additionally, efflux pump inhibitor ABI-PP cannot inhibit AcrB-Hi, while it inhibits the efflux ability of AcrB-Ec completely. The wide OmpP2 outer membrane porin from *H. influenzae* leaks small and elongated antibiotics such as β-lactams and novobiocin back into the cells, making Hib cells sensitive to these antibiotics. OmpF was rendered from a crystal structure (PDB identification code 3POX) and the structure of OmpP2 is based on a homology model. EPI efflux pump inhibitor

We showed that AcrB-Hi is a relatively ancient efflux pump, with a similar antibiotic efflux range as its evolved colleague AcrB-Ec. Despite the similar drug range, differences were additionally observed: AcrB-Hi is a significantly weak bile salt exporter compared to AcrB-Ec. In addition, AcrB-Hi is not inhibited by the efflux pump inhibitor ABI-PP. Furthermore, we showed that outer membrane porin OmpP2 leaks β-lactams and novobiocin, providing a molecular explanation for the high sensitivity of *H. influenzae* to these antibiotics. The results are summarised in Fig. 4.

We found that ancestral RND transporter AcrB-Hi is relatively closely related to *P. aeruginosa* MexI and MexW. The MexGHI-OpmD system can export vanadium^28^ and several classes of antibiotics^43^. MexVW-OprM can export a wide range of antibiotics, including erythromycin and ethidium bromide^29^. As *H. influenzae* cells harbour merely one RND transporter, it can be argued that AcrB-Hi plays a physiological role besides being a MDR efflux pump. Being closely related to MexW, AcrB-Hi may be involved in the production of quorum sensing signal molecules^43^, indirectly accounting for pathogenicity and promotion of persistence and antibiotic resistance of other bacteria^44^. Additionally, the transporter causes a decreased susceptibility for macrolide antibiotics in clinical strains^14^.

We also found that AcrB-Hi has a similar antibiotic efflux spectrum as AcrB-Ec, despite these transporters being significantly phylogenetically unrelated within the analysed 393 RND efflux genes (Fig. 2, Supplementary Fig. 2, 3, Supplementary Data 1). In a previous study, the highest MIC values for *H. influenzae* cells were observed for crystal violet and erythromycin (4 and 2.5 µg mL^−1^, respectively)^40^, consistent to the high efflux activity of these compounds by AcrB-Hi (Table 1). Interestingly, in the same study, the MIC values for novobiocin were significantly lower (0.13 µg mL^−1^, and only two dilutions higher than *acrB* KO cells). Additionally, in the same study, the MIC for β-lactam carbenicillin was unaffected (0.08 µg mL^−1^) within *H. influenzae* cells when the *acrB* gene was disrupted^40^. Furthermore, MIC values for other β-lactams (e.g. for ampicillin and benzylpenicillin) are significantly low (0.02 and 0.06 µg mL^−1^, respectively) for *H. influenzae* cells^41^. The quantitative MIC order for these compounds for intrinsically AcrB-Hi-expressing *H. influenzae* cells is therefore CV > EM≫NOV > β-lactams. On the other hand, in our study we observe high MIC values for novobiocin and β-lactams when AcrB-Hi was expressed in *E. coli* cells, which were even higher than for erythromycin and crystal violet (Table 1). For these compounds the MIC order is reversed to β-lactams = NOV > EM > CV. We provide a molecular explanation by the presence of OmpP2 in the OM of *H. influenzae* cells. This porin is naturally not present in *E. coli* cells. When we expressed OmpP2 in *ompF*-deficient *E. coli* cells, we saw a significant decrease in MICs for β-lactams (including cloxacillin and methicillin) and novobiocin (Table 3, Supplementary Fig. 8). At the same time, we saw no effect for erythromycin, and somewhat effect for crystal violet (Table 3). Therefore, we can directly explain the sensitivity of the Hib cells to β-lactams and novobiocin, as these compounds traverse through the porin channel back into the cells. Similarly, mutations in the OmpC porin in *E. coli* caused a higher sensitivity to β-lactams, as these mutations increased the width of the pore^45^. As the MICs were unaffected for high-molecular-mass drug erythromycin, it is reasonable to argue that novobiocin despite its high-molecular mass is a rod-like elongated molecule, while erythromycin is a bulkier sphere-like molecule^46^, hence a slow influx rate. In a previous study^41^, the OmpP2 molecular weight exclusion limit was estimated to be about 1400 Da. To compare, *E. coli* OmpF has an exclusion limit estimated to be 600 Da^47^. Interestingly, despite the high exclusion limit of OmpP2, small cationic compounds such as ethidium bromide were unaffected by the expression of OmpP2 (Table 3), which corroborates the high MIC of ethidium bromide for Hib cells (1.25 µg mL^−1^, and three dilutions higher than the *acrB* KO cells)^40^. OmpP2 seems to be a semi-selective channel for neutral compounds; however, it must be noted that the porin also impacted the MIC for cationic crystal violet (Table 3).

Additionally, we found that AcrB-Hi was able to export bile salts only weakly, while AcrB-Ec is a strong bile salt pump^17^ (Table 2, Supplementary Fig. 5). As *E. coli* is an enteric bacterium, it is essential for the organism to export bile salts^48^; however, *H. influenzae* is naturally not residing in a bile salts-rich environment. We also found that aminoglycoside kanamycin, third-generation cephalosporin ceftriaxone, and monobactam aztreonam were not exported. Third-generation cephalosporins such as ceftriaxone or cefotaxime are recommended antibiotics to treat *H. influenzae* infections—even when β-lactamases are identified in the strain^5^—and the inability of AcrB-Hi to export these drugs (Table 1) in addition to the high permeability of OmpP2 (Table 3) explains the effectiveness of these β-lactams. Monobactam aztreonam (used to treat *E. coli* and *P. aeruginosa* infections^5^) is exported by AcrD-Ec, but not by AcrB-Ec nor AcrB-Hi^22^. Figure 2 shows that AcrD-Ec is located in a different cluster than AcrB-Ec, which include MexB and MexY. Aminoglycosides are exported by AcrD-Ec and MexY, but not by AcrB-Ec, MexB and AcrB-Hi^49,50^. Figure 2 shows that MexB, MexY, and AcrD-Ec share the same cluster; however, MexB is located in a separate sub-cluster. *A. baumannii* AdeB from another earlier distinct cluster (Fig. 2) is also involved in aminoglycoside resistance^51^. It is interesting that AcrB-Hi from an ancestral cluster cannot export aminoglycosides, while some RND transporters from adapted clusters (MexY, AcrD-Ec) can, while other RND transporters (MexB, AcrB-Ec) cannot.

**Fig. 5:**
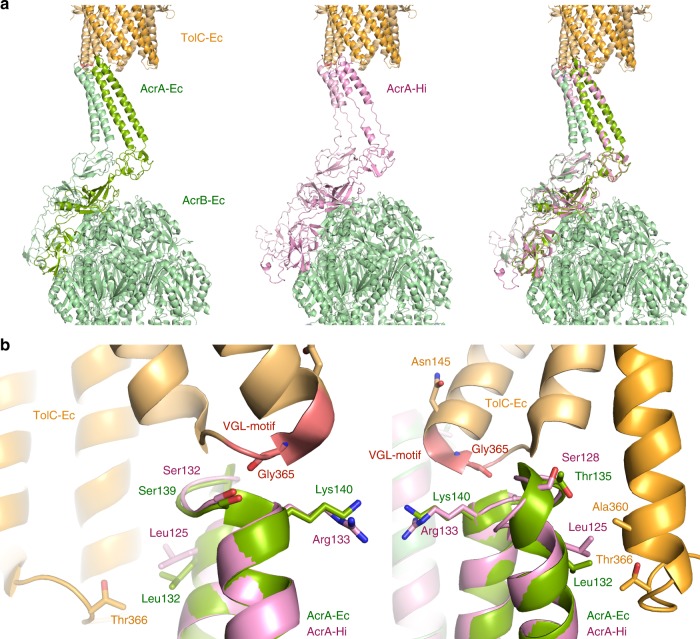
AcrA-Hi modelled in the tripartite structure of AcrAB-TolC. The homology model of AcrA-Hi is shown in pink and the actual AcrAB-Ec cryo-EM structure is shown in green. TolC-Ec is shown in orange. The TolC conserved VGL-motif is shown in red. **a** The AcrAB-TolC complex (shown are two out of the total six AcrA chains). The left image is the AcrAB-TolC-Ec structure (PDB identification code 5O66). The centre image is the homology model of AcrA-Hi modelled on AcrB-Ec and TolC-Ec. AcrA-Hi is depicted in pink. Visible are the elongated chains, rather than a helix conformation. In AcrA-Ec, the two helices are Pro98-Leu133 (36 residues) and Ser139- Ala172 (34 residues). In the AcrA-Hi model, the helices are Ser99-Leu125 (27 residues) and Gln134-Ile158 (25 residues), a significantly shorter region than for AcrA-Ec. The shorter AcrA-Hi is functional with TolC-Ec, despite the shorter arms. **b** Interactions of AcrA-Ec (green) or AcrA-Hi (pink) with TolC-Ec (orange). The AcrA-Ec residues interacting with TolC-Ec are depicted as sticks. The backbone of Gly365 in TolC-Ec is interacting with AcrA-Ec Ser139 and the backbone of Lys140, or with the corresponding AcrA-Hi Ser132 and the backbone of Arg133. Conserved interacting residues among AcrA-Ec and AcrA-Hi are Leu132/Leu125 and Ser139/Ser132, respectively, and similar residues are Lys140/Arg133 (positively charged) and Thr135/Ser128 (polar). These conserved residues can explain why AcrAB-Hi is functional with TolC-Ec

We were only able to express functional AcrAB-Hi when the upstream AcrR-Hi gene was added to the construct. The presence of AcrR-Hi could have influenced the MIC data, as the repressor would have bound to the DNA if no AcrR substrate was present. However, we expressed AcrAB-Hi in the presence of bile salts (Supplementary Fig. 4) and found that the expression level of AcrB-Hi was similar to that of AcrB-Ec. We are not sure why the removal of *acrR*-Hi from the construct resulted in non-functional *acrAB*-constructs; however, the same phenomenon was observed previously^13^. In addition, AcrAB-Hi was functional with TolC-Ec. We investigated the AcrA/TolC interactions by homology modelling based on the tripartite structure of AcrAB-TolC^52^. Initial alignment of AcrA-Hi (382 amino acids) with AcrA-Ec (397 amino acids) results in 24% identity, 43% similarity and 15% gaps. Figure 5 shows the homology model of AcrA-Hi modelled on the tripartite structure of *E. coli* AcrAB-TolC^52^. In the model, the helices of the arms of AcrA-Hi (about 26 residues) are significantly shorter than AcrA-Ec (about 35 residues). However, residues interacting with TolC-Ec are conserved (Leu125 and Ser132) or similar (Arg133 and Ser128) in AcrA-Hi compared to AcrA-Ec. These residues can explain by tip−tip interactions the functional AcrAB-Hi when coupled with TolC-Ec. We cannot be completely sure whether or not the substitution of TolC-Hi with TolC-Ec slightly altered the drug susceptibility. However, the active pumping of the drugs by AcrB-Hi resulted in the elevated MICs. This indicates the substrate specificity to these tested compounds of the efflux pump AcrB-Hi itself.

Furthermore, AcrB-Hi was completely uninhibited by ABI-PP (Fig. 3). ABI-PP strongly inhibits AcrB-Ec and MexB^25^. ABI-PP cannot inhibit MexY, as MexY has a bulky tryptophan in the Phe-rich pit of the distal binding pocket, sterically hindering the binding of ABI-PP^25^. As for AcrB-Hi, the lack of a Phe-rich pit (Fig. 1, Supplementary Fig. 1) probably prevents the strong required binding to the inhibitor’s aromatic moieties. Homology modelling of the AcrB-Hi hydrophobic trap shows no steric hindrance (Supplementary Fig. 6). However, we cannot be certain how the hydrophobic pit of AcrB-Hi actually is. The addition of the Phe-residues by evolution may have contributed to the increase of export efficiency by AcrB-Ec, as aromatic compounds such as novobiocin and rhodamine 6G are significantly more efficiently exported by AcrB-Ec than AcrB-Hi (Table 1, Supplementary Fig. 5), and Phe-substitutions in AcrB-Ec significantly decrease the MIC for rhodamine 6G (Supplementary Table 2). The Phe-rich pit is nonetheless a disadvantage of these improved RND efflux pumps, since they can be inhibited by current EPIs^53^. It additionally points to a potential weakness in these presently developed EPIs^25,35,54^, as genomic point mutations causing Phe-substitution may render the EPIs ineffective.

We hypothesise based on the phylogenetic analysis and drug export ability data that multidrug recognition is not an evolutionarily acquired ability present only in the latest evolved efflux pumps. Supplementary Table 3 shows the residues in the pockets and the hydrophobic trap of several RND transporters interacting with substrates (minocycline, doxorubicin, and erythromycin) according to the AcrB-Ec crystal structures^36,37^. Some residues are conserved or similar between transporters (but not for all transporters) and certain residues within the same phylogenetic cluster tend to be more similar. AcrB-Ec and AcrB-Hi also show conserved residues in the binding pockets; however, there are also several differences (Supplementary Table 3). We hypothesised before that drugs may be occluded in the distal binding pocket, and not bound strongly to specific residues, oscillating and moving within the voluminous binding pocket, before being pushed out by peristaltic motion^16,55^. Multidrug recognition may not be dependent on recognition within the distal binding pocket. We hypothesise that compounds are initially selected at the entrances at the lipid bilayer, periplasm, and central cavity, based on their physicochemical properties. Supplementary Table 3 suggests that the Phe-rich pit is present in all three AcrB-Ec, AcrF-Ec and AdeJ (from *Acinetobacter baumannii*) belonging to the same cluster. Transporters in clusters genetically further distanced show fewer Phe-residues. We hypothesise that the Phe-rich hydrophobic trap is acquired by evolution. The inhibition by ABI-PP might be an unexpected evolutionary result for the AcrB-Ec efflux pump. We note that this is a hypothesis and it would be interesting for future research to study the differences between other RND transporters in more detail.

To conclude, a recent serious problem in Hib chemotherapy are β-lactam-resistant strains, which are accounted for by β-lactamases (in BLPAR strains) or mutations in PBPs (in BLNAR strains). However, we emphasise that the overexpression of the AcrB-Hi pump in clinical strains is a potential risk for the development of MDR Hib pathogenic strains, as we show the wide substrate specificity of this pump. Furthermore, the loss of porin expression or porin mutations are well-known factors in acquired drug resistance in many organisms including *E. coli* and *P. aeruginosa*^56,57^. Mutations causing a loss in expression of OmpP2 or a narrowing of the porin could potentially also turn sensitive Hib strains into drug-resistant clinically relevant strains. In addition, we suggest that ancestral efflux pumps extrude similar hydrophobic and amphiphilic toxic compounds and that these nonspecific and less-efficient transporters have evolved into specific, more efficient transporters, and that multidrug recognition itself is not an evolutionarily acquired ability. Our knowledge on pharmacokinetics and the molecular defence mechanism by multidrug efflux systems including its evolution is crucial for the development of novel antibiotics.

## Methods

### Bacterial strains and growth conditions

The *E. coli* MG1655 strain^58^ was used as wild-type strain and ∆*acrB* (NKE96)^59^ and ∆*acrAB* were derived from MG1655. In addition, the *E. coli* C43(DE3) strain was used for the OmpP2-related minimal inhibition concentration experiments. Gene deletions (∆*acrB*, ∆*acrAB* and ∆*acrB*∆*ompF*) were performed according to the method of Datsenko and Wanner, with recombination between short homologous DNA regions catalysed by phage **λ** Red recombinase^60^. The drug resistance markers were eliminated using plasmid pCP20^60^. The bacterial strains were grown at 37 °C in Luria−Bertani broth^61^.

### Protein analysis and phylogenetic tree

Homologous RND transporters genes were identified by homology search of *acrB*-Hi and *acrB*-Ec genes within the genome of interest from the NCBI genome database (GenBank) by GENETYX software. An overview of the analysed genes and their sequences can be found in Supplementary Data 1. Multiple sequence alignment was executed by Clustal Omega from EMBL-EBI^62^ and phylogenetic trees were visualised with iTOL^63^. Homology models were created using SWISS-MODEL^64^.

### Amplification and cloning

Three adjacent genes HI0893 (*acrR*), HI0894 (*acrA*) and HI0895 (*acrB*) from the *Haemophilus influenzae* strain Rd KW20 (ATCC 51907) genome were amplified together using primers, including a C-terminal 6-histidine tag. pBAD33 vectors were digested by *Sal*I and *Hin*dIII restriction enzymes (NEB) and the insert was cloned into pBAD33 vectors by InFusion (TaKaRa), resulting in pBAD33acrRABHiT (10.3 kbp). The *ompP2* gene (HI0139) was amplified using primers and cloned by InFusion (TaKaRa) into *Xba*I- and *Hin*dIII-digested pET26b(+) vectors (Novagen, Merck), resulting in pET26b(+)ompP2 (6.4 kbp). All constructs were confirmed by agarose gel and nucleotide sequencing (FASMAC, Japan). Amplification primers can be found in Supplementary Table 1.

### Site-directed mutagenesis

The plasmid pBAD33acrB (pBAD33 carrying the *acrB*-gene from *E. coli* MG1655, including a C-terminal hexahistidine-tag) was used for the site-directed mutagenesis. Primers were used to introduce the mutations by polymerase chain reaction (PCR). The mutations were confirmed by nucleotide sequencing (FASMAC, Japan). Plasmids were transformed in *acrB-*deficient MG1655 *E. coli* cells.

### Drug susceptibility by MIC

The MIC values were determined using growth ability in liquid LB or on LB agar plates supplemented with substrates, in a series of dilutions. Cell cultures of MG1655∆*acrB*, MG1655∆*acrAB*, C43(DE3)∆*acrB*, C43(DE3)∆*acrAB* and C43(DE3)∆*acrB∆ompF* cells harbouring the plasmid of interest (pBAD33acrRABHiT, pBAD33acrBhis and/or pET26b(+)ompP2) were grown overnight, and liquid cultures (supplemented with 10 mM arabinose, or both 10 mM arabinose and 0.1 mM IPTG) were incubated, shaken at 37 °C and OD_600 nm_ readings were done using the Infinite M Nano (200 PRO series, Tecan). ABI-PP concentrations were 64 µg mL^−1^. For LB agar MIC determination experiments, overnight grown cells were stamped on LB agar plates (supplemented with 10 mM arabinose) and incubated at 37 °C overnight. MIC values were defined as the lowest drug concentrations at which the cells were no longer viable. AcrB expression levels were measured by western blotting using an iBind western system (ThermoFisher), with an anti-his-tag mAb (MBL) as the primary and anti-mouse IgG HRP-linked whole Ab from sheep (GE Healthcare) as the secondary antibody. AcrB-Ec and AcrB-Hi were expressed in *E. coli* cells in LB medium supplemented with 10 mM arabinose, with and without the addition of 1000 µg mL^−1^ bile salts.

### Statistics and reproducibility

MIC experiments and the inhibition ability experiments were repeated multiple times to validate the reproducibility. Phylogenetic analysis and clustering was based on 393 different homologous RND-type transporters and multiple sequence alignment was performed several times.

### Reporting summary

Further information on research design is available in the Nature Research Reporting Summary linked to this article.

## Supplementary information

Supplementary Information

Description of Additional Supplementary Files

Supplementary Data 1

Reporting Summary

## Data Availability

Data are available in this published article itself and the supporting figures and tables are available as Supplementary Information files. The table with the homologous RND genes can be found as Supplementary Data 1. Other data that support the findings of this study are available from the corresponding author upon request.
