# Supplementary material for: Phylogenetic and functional characterisation of the Haemophilus influenzae multidrug efflux pump AcrB
Source: Commun Biol. 2019 Sep 13;2:340. doi: 10.1038/s42003-019-0564-6 (PMC6744504; doi:10.1038/s42003-019-0564-6)
Supplement: Supplementary file 1 — Supplementary Information [file 42003_2019_564_MOESM1_ESM.pdf]

Supplementary Figures

Score	Expect	Method	Identities	Positives	Gaps
483 bits(1242)	1e-157	Compositional matrix adjust.	329/1028(32%)	540/1028(52%)	44/1028(4%)

```
Query  11    DIFIRRPVLAVSISLLMIILGLQAISKLAVREYPKMTTTVITVSTAYPGADANLIQAFVT  70
             + FI RP+ A  I++++++ G  AI KL V +YP +    +T+S +YPGADA  +Q  VT
Sbjct  3     NFFIDRPIFAWVIAIIIMLAGGLAILKLPVAQYPTIAPPAVTISASYPGADAKTVQDTVT  62

Query  71    SKLEESIAQADNIDYMSSTSAPSSST-ITIKMKLNTDPAGALADVLAKVNAVKSALPNGI  129
               +E+++    DN+ YMSS S   + +  IT+  +  TD    A   V  K+       LP  +
Sbjct  63    QVIEQNMNGIDNLMYMSSNSDSTGTVQITLTFESGTDADIAQVQVQNKLQLAMPLLPQEV  122

Query  130   EDPSVS-SSSGGSGIMYISF--RSKKLDSSQVTDYINRVVKPQFFTIEGVAEVQVFGAAE  186
             +   VS    S  S +M +          +     ++DY+    +K         GV  +VQ+FG+ +
Sbjct  123   QQQGVSVEKSSSSFLMVVGVINTDGTMTQEDISDYVAANMKDAISRTSGVGDVQLFGS-Q  181

Query  187   YALRIWLDPQKMAAQNLSVPTVMSALSANNVQTAAGNDNGYYVSYRNKV------ETTTK  240
             YA+RIW++P ++     L+    V++A+ A N Q AAG    G          ++        +T
Sbjct  182   YAMRIWMNPNELNKFQLTPVDVITAIKAQNAQVAAGQLGGTPPVKGQQLNASIIAQTRLT  241

Query  241   SVEQLSNLIISSNGD-DLVRLRDIATVELNKENDNSRATANGAESVVLAINPTSTANPLT  299
             S E+    +++   N D    V LRD+A +EL   EN  +   A   NG   +   L I    + AN L
Sbjct  242   STEEFGKILLKVNQDGSRVLLRDVAKIELGGENYDIIAEFNGQPASGLGIKLATGANALD  301

Query  300   VAEKIRPLYESIKTQLPDSMESDILYDRTIAINSSIHEVIKTIGEATLIVLVVILMFIGS  359
              A  IR      ++    P  ++     YD T   +  SIHEV+KT+ EA ++V +V+ +F+ +
Sbjct  302   TAAAIRAELAKMEPFFPSGLKIVYPYDTTPFVKISIHEVVKTLVEAIILVFLVMYLFLQN  361

Query  360   FRAILIPILAIPISLIGVLMLLQSFNFSINLMTLLALILAIGLVVDDAIVVLENIDRHI-  418
             FRA LIP +A+P+ L+G    +L +F FSIN +T++   ++LAIGL+VDDAIVV+EN++R +
Sbjct  362   FRATLIPTIAVPVVLLGTFAVLAAFGFSINTLTMFGMVLAIGLLVDDAIVVVENVERVMA  421

Query  419   KAGETPPFRAAIIGTREIAVPVISMTIALIAVYSPMALMGGITGTLFKEFALTLAGAVFIS  478
             + G   P   A        +I    ++ + + L AV+ PMA   GG TG ++++F++T+  A+ +S
Sbjct  422   EEGLPPKEATRKSMGQIQGALVGIAMVLSAVFVPMAFFGGSTGAIYRQFSITIVSAMALS  481

Query  479   GVVALTLSPMMSSKLLKSNAKPTWMEERVEHTLGKVNRVYE----YMLDLV---MLNRKS  531
              +VAL L+P + + +LK   AK     E + +    G  NR++E      +  D V     + +
Sbjct  482   VLVALILTPALCATMLKPIAKGDHGEGK-KGFFGWFNRMFEKSTHHYTDSVGGILRSTGR  540

Query  532   MLAFAVVIFSTLPFLFNSLSSELTPNEDKGAFIAIGNAPSSVNVDYIQNAMQP----YM-  586
              L     ++I     + +LF   L S    P+ED+G F+ +      P+      +  Q   +       Y+
Sbjct  541   YLVLYLIIVVGMAYLFVRLPSSFLPDEDQGVFMTMVQLPAGATQERTQKVLNEVTHYYLT  600

Query  587   --KNVMETPEVSFGMSIAGAPTSNSSLNIITLKDWKER---SRKQSAIMNEINEKAKSIP  641
               KN  +E+       G    AG      N+ +   ++LKDW +R          K   AI              I
Sbjct  601   KEKNNVESVFAVNGFGFAGR-GQNTGIAFVSLKDWADRPGEENKVEAITMRATRAFSQIK  659

Query  642   EVSVSAFNIP---EIDTGEQGPPVSIVLKTAQDYKSLANTAEKFLS-AMKASGKFIYTNL  697
             +   V AFN+P     E+ T    G     ++ +     ++ L       + L+ A K
Sbjct  660   DAMVFAFNLPAIVELGTA-TGFDFELIDQAGLGHEKLTQARNQLLAEAAKHPDMLTSVRP  718

Query  698   DLTYDTAQMTISVDKEKAGTYGITMQQISNTLGSFLSGATVTRVDVDGRAYKVISQVKRD  757
             +    DT Q   I  +D+EKA     G+++   I+ TLG+     G+ V        GR   KV      +
Sbjct  719   NGLEDTPQFKIDIDQEKAQALGVSINDINTTLGAAWGGSYVNDFIDRGRVKKVYVMSEAK  778

Query  758   DRLSPESFQNYYLTASNGQSVPLSSVISMKLETQPTSLPRFSQLNSAEISAVPMPGISSG  817
              R+  P+     ++Y+  A++GQ  VP  S+    S  +  E       L  R++  L  S  EI        PG  S+G
Sbjct  779   YRMLPDDIGDWYVRAADGQMVPFSAFSSSRWEYGSPRLERYNGLPSMEILGQAAPGKSTG  838

Query  818   DAIAWLQQQATDNLPQGYTFDFKSEARQLVQEGNALAVTFALAVIIIFLVLAIQFESIRD  877
             +A++   ++Q  A++    LP  G    +D++     + Q       GN        +A+++I++FL  LA    +ES
Sbjct  839   EAMELMEQLAS-KLPTGVGYDWTGMSYQERLSGNQAPSLYAISLIVVFLCLAALYESWSI  897

Query  878   PMVIMISVPLAVSGALVSLNILSFFSIAGTTLNIYSQVGLITLVGLITKHGILMCEVAKE  937
             P    +M+  VPL  V  GAL++          +    G  T  ++Y  QVGL+T  +GL    K+  IL+  E  AK+
Sbjct  898   PFSVMLVVPLGVIGALLAA------TFRGLTNDVYFQVGLLTTIGLSAKNAILIVEFAKD  951

Query  938   EQLNHGKTRIEAITHAAKVRLRPILMTTAAMVAGLIPLLYATGAGAVSRFSIGIVIVAGL  997
                   GK   IEA     A  ++RLRPILMT+   A  +  G++PL+ +  +TGAG+  ++ ++  ++G   ++ G+
Sbjct  952   LMDKEGKGLIEATLDAVRMRLRPILMTSLAFILGVMPLVISTGAGSGAQNAVGTGVMGGM  1011

Query  998   SIGTIFTL  1005
                T+  +
Sbjct  1012  VTATVLAI  1019
```

Supplementary Figure 1. Amino acid sequence alignment of AcrB-Hi and AcrB-Ec.

AcrB-Hi is shown as the top sequence, and AcrB-Ec is shown as the bottom sequence. Red indicates the Phe-rich pit of AcrB-Ec and the corresponding residues in AcrB-Hi.

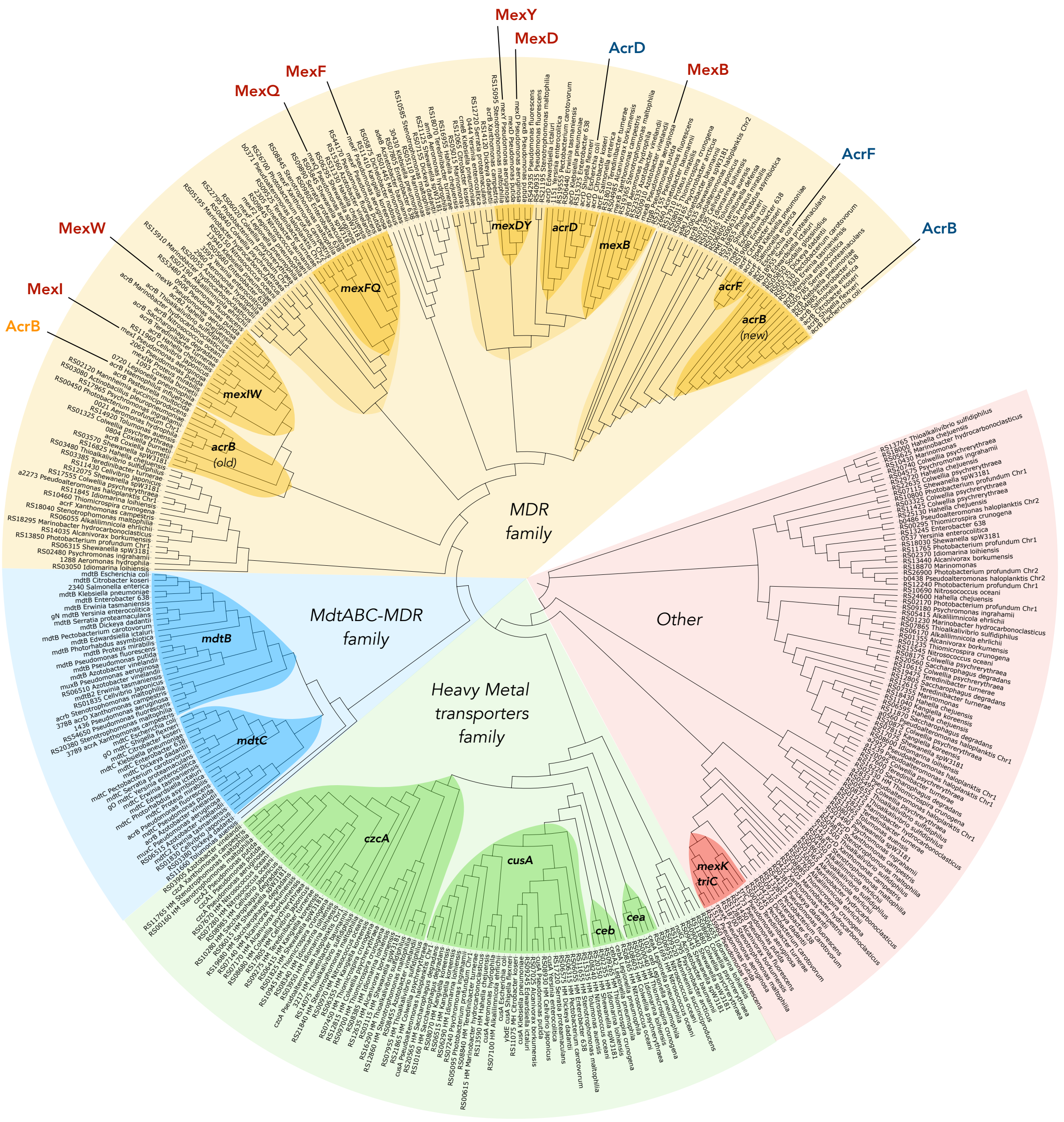

Supplementary Figure 2. Phylogenetic tree of 393 *acrB*-Ec and *acrB*-Hi homologous genes from 53 gammaproteobacteria.

Transporters are classified as multi-drug resistant (MDR, yellow), MdtBC-like MDR (blue), heavy metal (green) and other (red). Darker regions show clusters and sub-clusters of RND transporters, with annotations provided (such as *cusA*, *czcA*, *mdtB*, *mdtC*, *acrB*, *mexB*, etc). Gene names are either unidentified, unnamed genes, genes for which the function has been experimentally verified, or for which the function has been estimated based on sequence homology.

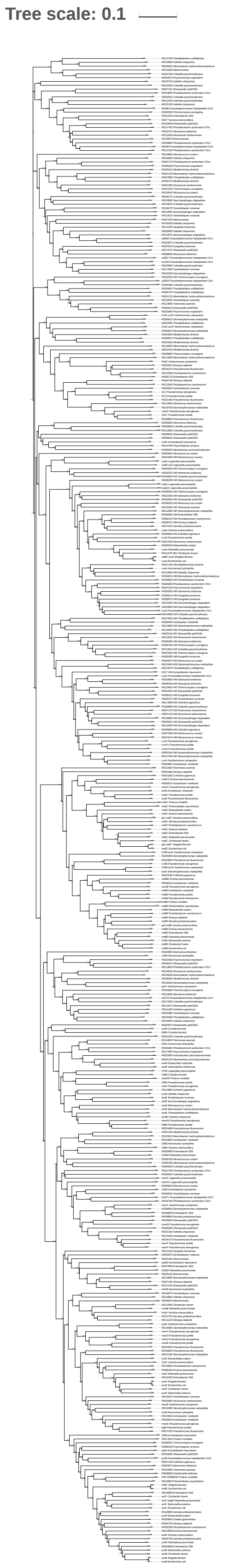

Supplementary Figure 3. Multiple sequence alignment results shown in a phylogenetic tree provided with branch lengths.

Gene names are either unidentified, unnamed genes, genes for which the function has been experimentally verified, or for which the function has been estimated based on sequence homology.

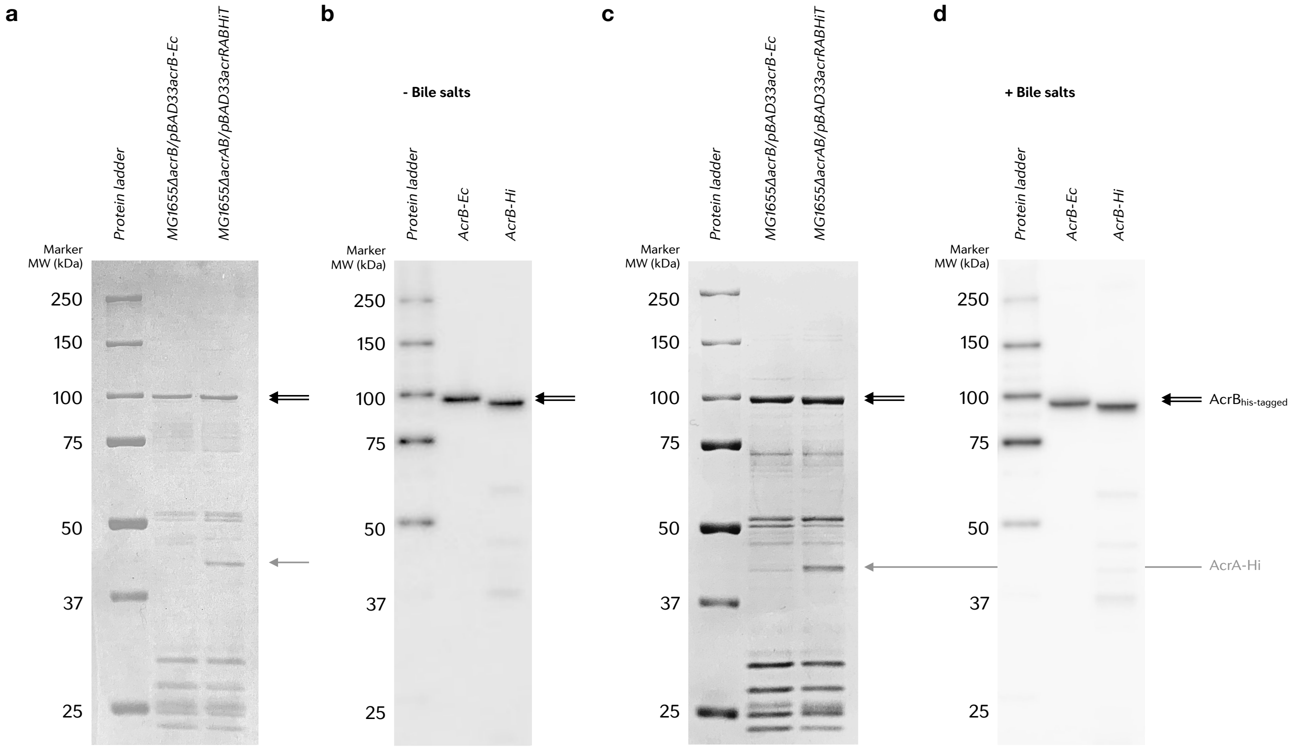

Supplementary Figure 4. Expression tests for AcrB-Ec and AcrB-Hi expressed in MG1655 *E. coli* cells.

a Coomassie-stained SDS-PAGE gel (10%) of membrane fraction of *E. coli* cells. Left shows AcrB-Ec expressing cells, right shows AcrB-Hi expressing cells. **b** Western blotting analysis of the same SDS-PAGE loaded membrane fractions. **c** Coomassie-stained SDS-PAGE gel of membrane fraction of *E. coli* cells in the presence of bile salts. **d** Western blotting analysis of the membrane fraction of *E. coli* cells grown in the presence of bile salts. For AcrB-Hi, faint lower molecular mass bands are slightly visible, pointing to either some sensitivity to protease activity after cell lysis, or translation initiation at internal methionines from the plasmid. AcrB-Ec and AcrB-Hi are expressed in a similar abundance. When bile salts are added, there is no change in the expression of AcrB-Hi (nor AcrB-Ec). AcrA-Ec has a molecular mass of about 40 kDa, AcrB-Ec and AcrB-Hi have a molecular mass of about 100 kDa. The same amount of protein is loaded on both lanes (the membrane fraction 2 µg). Bile salts were added to a final concentration of 1000 µg mL^{-1}.

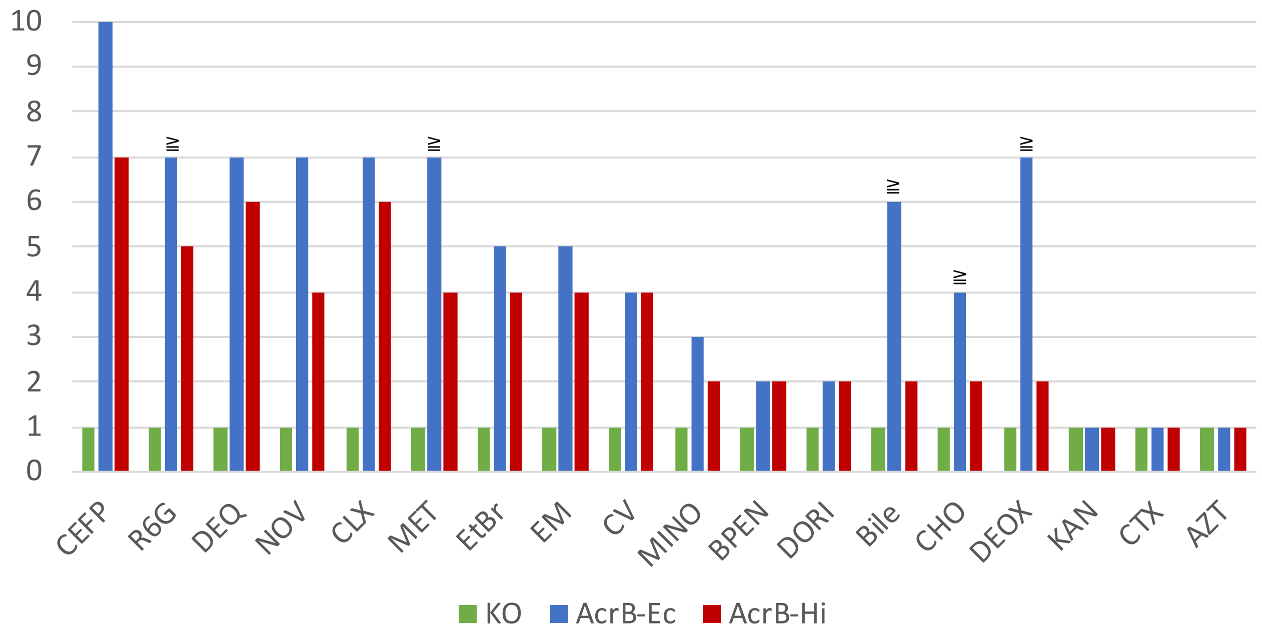

Supplementary Figure 5. Relative MIC dilution steps provided for all tested drugs.

Based on MICs seen in Tables 1-3, for *acrB*-KO cells (KO, green), AcrB-Ec (blue) and AcrB-Hi (red) expressing *E. coli* MG1655Δ*acr(A)B* cells. *EtBr* ethidium bromide, *R6G* Rhodamine6G, *CV* crystal violet, *MINO* minocycline, DEQ dequalinium, *KAN* kanamycin, *EM* erythromycin, *NOV* novobiocin, *CLX* cloxacillin, *BPEN* benzylpenicillin, *MET* methicillin, *CEFP* cefcapene pivoxil, *CTX* ceftriaxone, *DORI* doripenem, *AZT* aztreonam, *CHO* cholic acid, *DEOX* deoxycholic acid. The equal-to-or-larger mark (≧) indicates that the relative MICs could be even higher, as the tested MICs reached their limit.

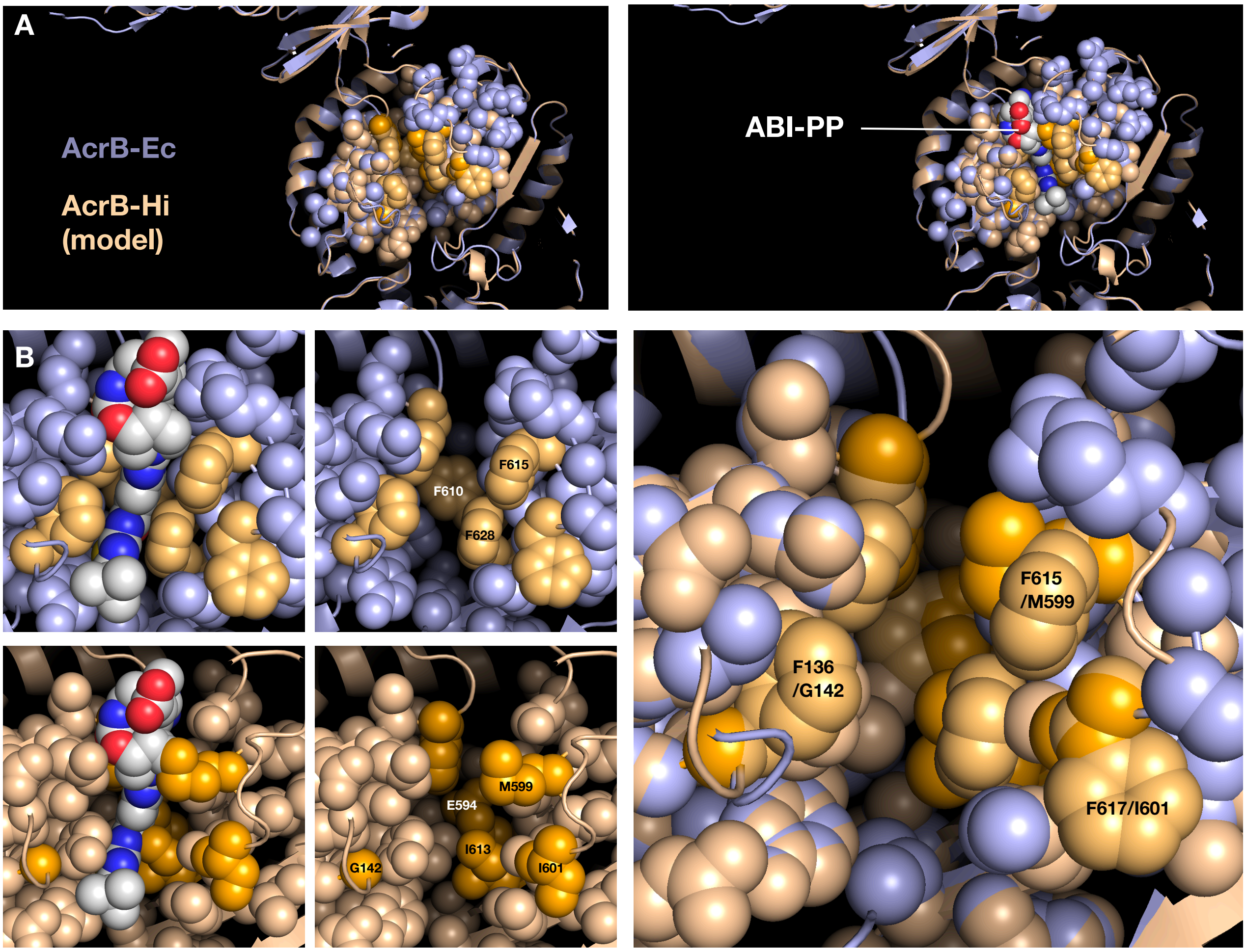

Supplementary Figure 6. Homology model of the hydrophobic pit of AcrB-Hi.

a AcrB-Ec (light blue) and AcrB-Hi (light orange) are superimposed with and without ABI-PP. Residues around the pit are shown in spheres. ABI-PP is shown in grey spheres. **b** A side view of the hydrophobic pit in both AcrB-Ec (top, light blue) and AcrB-Hi (bottom, light orange). The most left images show ABI-PP superimposed. The image on the right is a superimposed image of the pit of both AcrB-Ec and AcrB-Hi. The darker orange side chains show the Phe-residues of AcrB-Ec, or the corresponding residues in AcrB-Hi. The model was based on PDF identification code 3W9H.

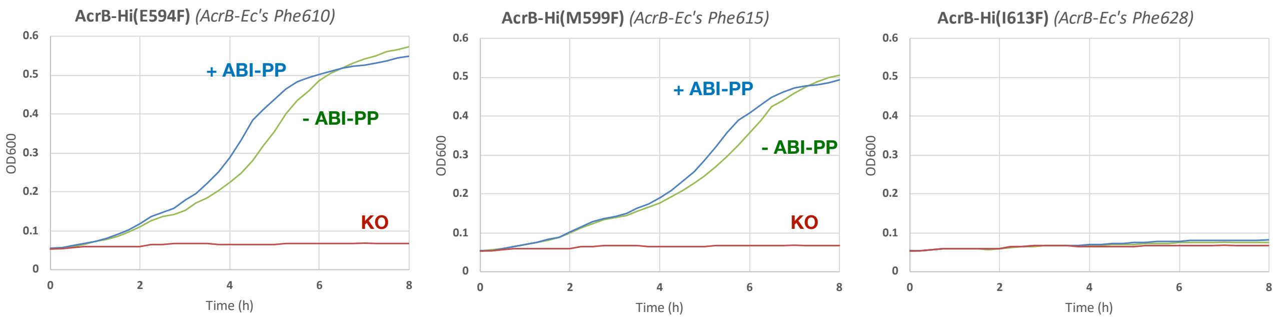

Supplementary Figure 7. The inhibition by ABI-PP of hydrophobic trap mutants of AcrB-Hi.

Growth curves of AcrB-Hi mutant expressing *E. coli* cells.Four mutations were tested, namely, Glu594Phe (left), Met599Phe (middle) and Ile613Phe (right). Depicted are the mutant expressing cells without ABI-PP (green), with the addition of ABI-PP (blue) and *acrB*-KO cells (red). All erythromycin concentrations were 16 µg mL^{-1} and ABI-PP concentrations were 64 µg mL^{-1}. Trying higher concentration of EM resulted in growth curves close to KO cells.

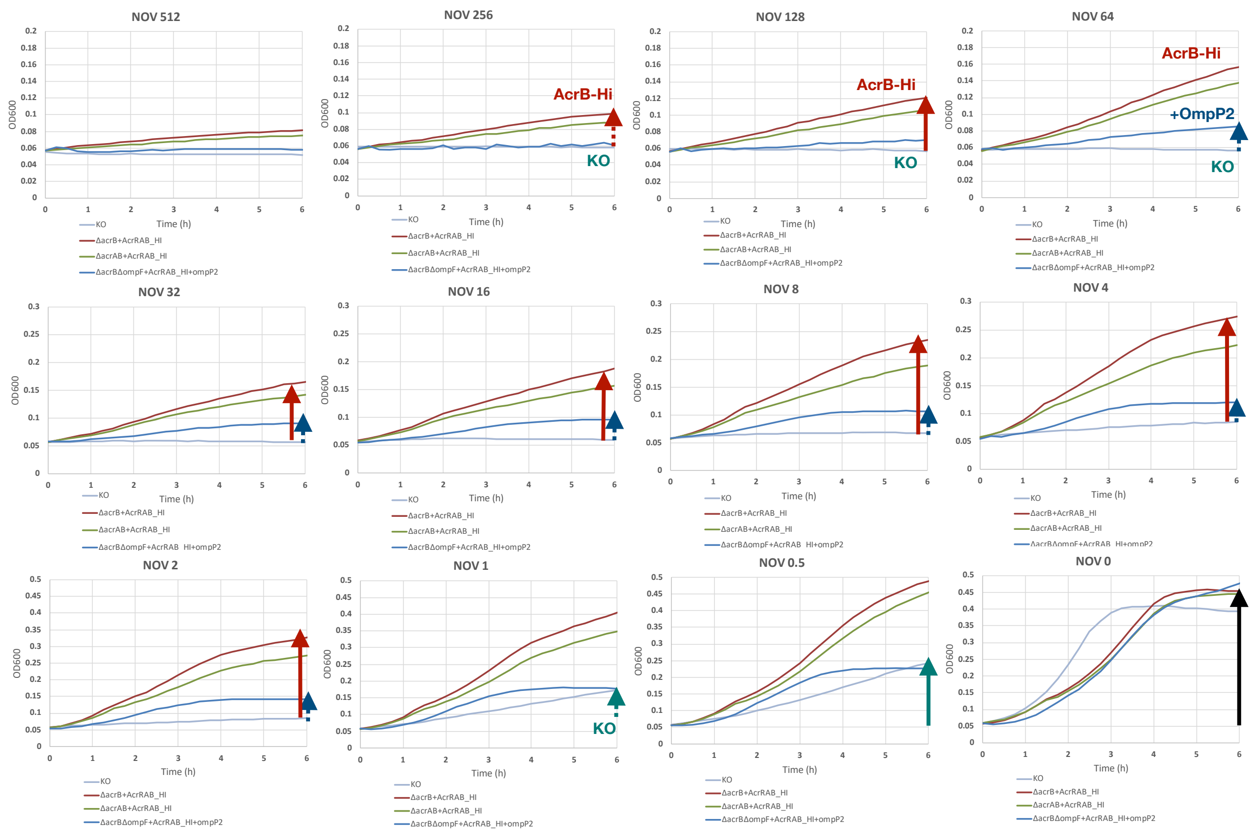

Supplementary Figure 8. The effect of novobiocin on OmpP2-expressing cells.

Growth curves for *acrB*- and *ompF*-KO (light blue), AcrRAB-Hi (green, red), and AcrRAB- and OmpP2 (dark blue) expressing *E. coli* C43(DE3) cells. The cells are effected by the expression of OmpP2 under novobiocin exposure for all tested concentrations.

Supplementary Tables

Primer name	Sequence
AcrRAB-Hi Forward	ATCCTCTAGAGTCGACGCTCATTCATTTTTGAGCAATTATTGCTCCTTATTT
AcrRAB-Hi (his6-tagged) Reverse	ATGCCTGCAGGTCGACTTA**ATGGTGATGGTGATGGTG**GGTTGTTTTATTTT
OmpP2 Forward	GTGCGGCCGCAAGCTTGCGAATCTTTCGATTCGCCTTGCTT
OmpP2 Reverse	ACAATTCCCCTCTAGAGGAGAAAAGTTCAATCATAGATAGT

Supplementary Table 1. Primers used for the amplification of *acrRAB* and *ompP2* from *H. influenzae.* These amplified segments were used for cloning in the designated vectors pBAD33 or pET26b(+).

		Strain	Plasmid	Minimal inhibitory concentration (MIC, µg/mL)					
				**EtBr**	**R6G**	**EM**	**MET**	**OXA**	**PIP**
	WT	MG1655ΔacrB	pBAD33acrBhis	1024	4096	256	512	256	2
	KO	MG1655ΔacrB	*pBAD33*	8	32	8	4	1	0.125
Pit		MG1655ΔacrB	pBAD33acrB(F136I)his	*1024*	**1024**	*128*	*1024*	*128*	*2*
		MG1655ΔacrB	pBAD33acrB(F136G)his	**256**	**128**	**64**	*256*	**32**	*1*
		MG1655ΔacrB	pBAD33acrB(F628I)his	*512*	**256**	*128*	*256*	**32**	*1*
		MG1655ΔacrB	pBAD33acrB(F136G+F628I)his	**128**	**64**	**16**	**64**	**4**	**0.25**
		MG1655ΔacrB	pBAD33acrB(F610E)his	*512*	**1024**	128	*256*	*128*	*1*
		MG1655ΔacrB	pBAD33acrB(F610A)his	**128**	**256**	**32**	**128**	**32**	**0.5**
Loop		MG1655ΔacrB	pBAD33acrB(F615M)his	*512*	*2048*	*128*	*256*	*128*	*1*
		MG1655ΔacrB	pBAD33acrB(F617I)his	*1024*	*4096*	*128*	*1024*	*128*	*1*
		MG1655ΔacrB	pBAD33acrB(F615M+F617I)his	*512*	*4096*	**64**	*256*	*128*	*0.5*

Supplementary Table 2. MIC data of several single and double AcrB-Ec hydrophobic pit mutations. Bold underlined shows an at least two dilution lowering in MIC, italic shows zero to one difference in MIC, compared to WT AcrB expressing cells. *EtBr* ethidium bromide, *R6G* Rhodamine6G, *EM* erythromycin, *MET* methicillin, *CEFP* cefcapene pivoxil, *CTX* ceftriaxone, *OXA* oxacillin, *PIP* piperacillin.

Cluster	Organism	Transporter	Comparison to AcrB-Ec			Residues																							
			Identity	Similar	Gaps	Hydrophobic trap						Distal Binding Pocket											Proximal Binding Pocket						
1	*Escherichia coli*	**AcrB**	100%	100%	0%	**F136**	**F178**	**F610**	**F615**	**F617**	**F628**	**S46**	**Q89**	**S128**	**E130**	**Q176**	**L177**	**S180**	**E273**	**N274**	**I277**	**V612**	**S79**	**T91**	**S134**	**S135**	**K292**	**L674**	**D681**
1	*Escherichia coli*	**AcrF**	78%	88%	0%								T					A			V								
1	*Acinetobacter baumannii*	**AdeJ**	58%	75%	2%							A	S	T	T		V	G	D		F			Q	A				N
2	*Escherichia coli*	**AcrD**	66%	80%	0%	N	Y		S	P		T	S	R	R	D	A			K	Y	T			D	T			
2	*Pseudomonas aeruginosa*	**MexB**	71%	84%	0%							Q	T	R	T		V		Q	D					K	N			
2	*Pseudomonas aeruginosa*	**MexD**	49%	67%	1%							T	E	Q			F			L	F	I	E	V	A	G	P		A
2	*Pseudomonas aeruginosa*	**MexY**	48%	67%	1%	I	W	Y		L			S	Y		E	T	A	S	E	F		K		D				
3	*Acinetobacter baumannii*	**AdeB**	49%	68%	1%			T	W				E	Q				A	Q	A	F	I				G	Q		S
4	*Haemophilus influenzae*	**AcrB**	32%	52%	4%	G or I		E	M	I	I		-		S		V	A			S	S			G		N	I	P
4	*Legionella pneumophila*	**AcrB**	32%	52%	3%	A	L		-	-	G	M	T	L	K	D	I	G	Q		S		T			T	T	-	A
4	*Pasteurella multocida*	**AcrB**	31%	52%	4%	G		M	-	-	V		-	T	S		I		S	S	S	I			G	T	N	Q	-
4	*Coxiella burnetii*	**AcrB**	30%	52%	4%	-	A	V	K				D	Q	G	E	V	A	D	S	S		I	K	M	E	N	-	E
4	*Photobacterium profundum*	**AcrB**	33%	53%	3%	A	Y		-	V	-	T	T	T	S	D		G	K	S		-	T		T	T	N	-	P

Supplementary Table 3. Comparison of aligned residues belonging to the hydrophobic trap, distal binding pocket and proximal binding pocket of several MDR RND transporters. Residues are based on the co-crystal structures of AcrB-Ec and their substrates, thought to interact with moieties of the drug molecules. Green shows a conservation compared to AcrB-Ec's residues, blue indicates a similar substitution and red shows a different amino acid substitution. A bar with a white background indicates that no aligned residue was found. Alignment was performed by BLAST. Cluster numbers are to indicate the transporters belonging to different clusters. Precise clusters can be found in Fig. 2 or Supplementary Fig. 2 and 3.
