# Supplementary material for: Phylogenetic and functional characterisation of the Haemophilus influenzae multidrug efflux pump AcrB
Source: Commun Biol. 2019 Sep 13;2:340. doi: 10.1038/s42003-019-0564-6 (PMC6744504; doi:10.1038/s42003-019-0564-6)
Supplement: Supplementary file 2 — Description of Additional Supplementary Files [file 42003_2019_564_MOESM2_ESM.docx]

**File:** Supplementary Data 1.xlsx

**Description:** Table of homologous and orthologues RND-type transporter genes from several gammaproteobacteria.
